# Adjunctive TNF Inhibition with Standard Treatment Enhances Bacterial Clearance in a Murine Model of Necrotic TB Granulomas

**DOI:** 10.1371/journal.pone.0039680

**Published:** 2012-06-27

**Authors:** Ciaran Skerry, Jamie Harper, Mariah Klunk, William R. Bishai, Sanjay K. Jain

**Affiliations:** 1 Center for Tuberculosis Research, Johns Hopkins University School of Medicine, Baltimore, Maryland, United States of America; 2 Center for Infection and Inflammation Imaging Research, Johns Hopkins University School of Medicine, Baltimore, Maryland, United States of America; 3 Department of Pediatrics, Johns Hopkins University School of Medicine, Baltimore, Maryland, United States of America; 4 KwaZulu-Natal Research Institute for Tuberculosis and HIV, Durban, South Africa; 5 Howard Hughes Medical Institute, Chevy Chase, Maryland, United States of America; Fundació Institut d’Investigació en Ciències de la Salut Germans Trias i Pujol. Universitat Autònoma de Barcelona. CIBERES, Spain

## Abstract

**Background:**

It has been hypothesized that early host-responses during TB treatment may paradoxically promote survival of persistent bacteria. We therefore evaluated whether adjunctive inhibition of tumor necrosis factor alpha (TNF-α)–a key cytokine in host responses against TB–could hasten bacterial clearance in a mouse strain that develops necrotic lesions in response to *Mycobacterium tuberculosis* infection.

**Methodology/Principal Findings:**

Six weeks after an aerosol infection, C3HeB/FeJ mice received standard TB treatment with or without adjunctive TNF inhibition (etanercept for the initial six weeks). Functional TNF-α levels and lung pathology were found to be reduced in the mice receiving etanercept. Compared to standard TB treatment, the addition of etanercept resulted in a significantly lower pulmonary bacterial burden, corresponding to the phase when a significant proportion of bacteria are multiplying slowly (p<0.0233). Finally, only 10.5% of mice receiving adjunctive etanercept versus 27.8% receiving standard TB treatment alone relapsed.

**Conclusion:**

This study provides proof-of-principle that modulation of TNF-α activity can hasten bacterial clearance during standard multi-drug TB treatment. Oral agents that modulate TNF-α should therefore be considered as adjunct therapies for shortening TB treatments. However, due to concerns of reactivation disease, additional studies need to be performed before TNF-α inhibitors are used for TB treatment in humans.

## Introduction

Recognizing that tuberculosis (TB) is still the leading cause of human death from a curable disease, the international health community has set an ambitious target to eliminate TB by 2050. Using mathematical modeling Dye *et al* have shown that the 2050 target cannot be achieved with current tools and requires a combination of new diagnostics, shorter TB drug regimens and new vaccines that can detect and treat both latent infection and active disease [1].

It has been hypothesized that early host-responses – inflammation, necrosis and subsequent hypoxia – during TB treatments may paradoxically promote survival of persistent bacteria [2]. In fact, adjunct corticosteroid use for the *initial* 6-weeks is helpful in certain forms of TB (meningitis, pleural TB) [3], though these data are less clear for pulmonary TB. Tumor necrosis factor alpha (TNF-α) is a cytokine that plays a central role in the host responses against TB, including formation of granulomas and containment of disease [4], [5], [6]. Although TNF-α inhibition leads to reactivation disease [7], [8], it has been hypothesized that adjunctive use of TNF-α inhibitors during TB treatments may paradoxically be beneficial [9]. This is plausible as TNF-α levels increase shortly after initiation of TB treatment [10], causing tissue destruction, creating a microenvironment which could favor bacterial survival. This hypothesis is supported by case reports and small series that demonstrate that adjunctive use of TNF-α inhibitors with TB treatments is beneficial [11], [12], [13], [14].

Pre-clinical evaluation of multi-drug TB treatments is well established in the mouse model of TB. However, necrosis and hypoxia, key pathological features of human TB lesions, postulated to favor survival of persistent bacteria, are lacking in conventional mouse strains. We therefore utilized C3HeB/FeJ mice, which develop well-organized [15] and hypoxic TB granulomas with central caseous necrosis [16], and evaluated whether adjunctive TNF-α inhibition combined with standard TB treatment could hasten bacterial clearance.

## Methods

### Ethics Statement

All animal procedures have been approved by the ethics committee of Johns Hopkins University.

### Animal Infections

Six-to-eight week old female C3HeB/FeJ (Jackson Laboratory) mice were aerosol infected with frozen titrated bacterial stocks of *Mycobacterium tuberculosis* H37Rv, using the Middlebrook Inhalation Exposure System (Glas-Col). Mice were sacrificed 1 day after infection and at 2, 4, 8, 10 and 12 weeks after starting TB treatments. Lungs and spleens were removed aseptically, homogenized and plated on Middlebrook 7H11 agar plates (Fisher, USA) to determine colony-forming unit (CFU). A minimum of 4 mice were used per group and for each time-point.

### Multi-drug TB Treatments

Treatment began 6 weeks after a low-dose aerosol infection. Mice were administered the standard TB regimen with Rifampin (10 mg/kg), Isoniazid (25 mg/kg) and Pyrazinamide (150 mg/kg) by gavage, 5 days per week for a total of 12 weeks. Pyrazinamide was administered only for the first 8 weeks, as is standard for TB treatment in humans [3]. Animals were injected intraperitoneally, twice weekly, with etanercept (Amgen, USA) (15 mg/kg) [17]. Etanercept - a soluble TNF receptor fusion molecule (sTNFR) was chosen for these experiments since the risk of reactivation is lower in patients receiving etanercept than in those receiving TNF antibodies, suggesting that sTNFR may be safer [18], [19].

### Relapse

Additional cohorts of mice were held for 12 weeks after cessation of treatment to assess for stable, relapse free cure. At this time, lungs and spleens were removed aseptically, homogenized and plated on Middlebrook 7H11 agar plates. The complete homogenate was plated across several plates, for each organ.

### Histopathology

To assess pulmonary inflammation and injury, entire mouse lungs were fixed by immersion in 10% (vol/vol) formalin, and following paraffin embedding, 4-µm longitudinal sections were cut, stained with hematoxylin and eosin. Images shown are representative of section obtained from 4 animals per group and for each time-point.

### Morphometric Analysis of Lung Tissue

To further evaluate the differences noted in lung pathology, morphometric analyses were performed on lung tissues obtained from the standard treatment (RHZ alone) and adjunctive etanercept (RHZ plus etanercept) arms using Image J software (NIH, USA). A minimum of three fields of view, obtained from four animals per group, for each time-point were used (12 fields per group for each time-point). Lung involvement was calculated as the percent of lung tissue occupied by lesions.

### ELISA

Cytokine concentrations were measured by sandwich ELISA in a 96 well plate pre-coated with capture antibodies (Abcam, USA). The plates were washed with Wash Buffer [PBS-Tween 20 (0.05% v/v)] and blocked using 300 µl of 10% (w/v) milk powder for 2 hours at room temperature before the addition of recombinant standards and samples and a 2 hour incubation at room temperature. Following incubation, plates were washed and detection antibody added for 1 hour at room temperature. Following this, streptavidin-HRP was added followed by 3,3′,5,5′ - tetramethylbenzidine (TMB) substrate and color allowed to develop in the dark. Plates were then read on a spectrophotometer at an optical density of 450 nm.

### TNF-α Activity

TNF-α activity was measured using the WEHI assay [20]. Lung homogenates were filter sterilized through a 0.22 µm filter before being diluted two-fold in sterile PBS and added, in triplicate, to a 96-well containing 2×10^5^ WEHI13-VAR cells (ATCC, USA). At the same time, a standard curve was prepared using 2-fold dilutions of recombinant murine TNF-α (Sigma-Aldrich, USA), ranging from 0 to 200 units per well. Following a 24 hour incubation with the lung homogenate or recombinant TNF-α at 37°C and 5% CO_2_, 100 µg of 3-(4,5-Dimethylthiazol-2-yl)-2,5-diphenyltetrazolium bromide was added to each well and incubated at 37°C, 5% CO_2_ for an additional 4 hours. Following the addition of DMSO, plates were read at 595 nm and activity calculated as a function of the standard curve. For neutralization assays samples were incubated for 60 minutes at 37°C with ten-fold dilutions of a goat anti-mouse TNF-α antibody (Abcam, USA). Triplicate, filter sterilized lung tissue homogenates were also tested for TNF-α using ELISA (as described above). Data was normalized to the total protein concentration of the tissue samples.

### Cytokine Analysis

Cytokine concentrations were determined by Bio-Plex Pro Assay (Bio-Rad, USA) according to the manufacturer’s instructions or ELISA (as described above). Four mice were used per group and for each time-point. Splenocytes were isolated by disaggregation of whole mouse spleens and seeded at a concentration of 1×10^6^ cells/ml in a total volume of 200 µl. After 72 h of incubation in the presence of heat-inactivated *M. tuberculosis* (H37Rv), culture supernatants were sampled for select cytokines. Dye labeled cytokine-specific antibodies were mixed with sample, washed and targeted with a biotinylated detection antibody. The resultant antibody sandwich was visualized using streptavidin-PE. Acquisition and analyses was then performed using the Luminex system. Results are presented as pg/ml of supernatant.

### Statistical Analysis

Statistical comparison between groups was performed using two tail Student’s *t* test in Prism 4 version 4.01 (GraphPad software, San Diego, CA). Data are presented on a logarithmic scale as mean ± standard deviation for CFU counts, except where stated.

## Results

### Adjunctive Etanercept Reduced Local TNF-α Activity in the Lung Tissues

**Figure 1:**
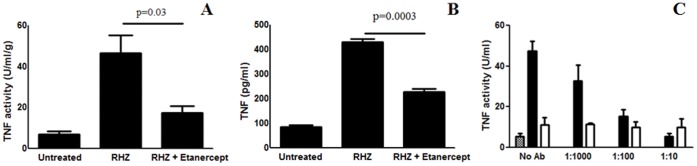
TNF-α activity in the lungs of treated mice. TNF-α activity was measured in lung homogenates obtained from *Mycobacterium tuberculosis* infected C3HeB/FeJ mice by WEHI assay (A) and ELISA (B). Levels for mice receiving no treatment (Untreated), standard TB treatment (RHZ) or with adjunctive etanercept (RHZ + Etanercept) were measured 7 days after the start of treatment. TNF-α activity increased with TB treatment and was abrogated by the administration of adjunctive etanercept. Moreover, to ensure that the TNF-α activity measured using the WEHI assay was indeed due to TNF-α and not other endogenous factors, lung homogenates from both the standard treatment (RHZ alone) and adjunctive etanercept (RHZ plus etanercept) arms were pre-incubated with increasing concentrations of a murine anti-TNF-α antibody and tested using the WEHI assay (C). Lung homogenates from the adjunctive etanercept arm (open bars) had consistently lower TNF-α activity which remained unaffected by pre-incubation with the neutralizing antibody. However, TNF-α activity in the lung homogenates from the standard treatment arm (filled bars) decreased in a dose-dependent manner with increasing concentrations of the neutralizing antibody, with activities at 100 or 10 fold dilutions reaching levels observed in the adjunctive etanercept arm. Analyses were performed on two-fold diluted lung homogenate from four mice per group. Data is normalized to the total protein concentration of the tissue samples and is presented as mean activity (U/ml/g) (±SD) in panels A, C and mean level (pg/ml) (±SD) in panel B.

Soluble TNF-α inhibitor, etanercept, was administered as an adjunct to standard TB treatment (RHZ) and bacterial burden measured in the lungs of treated animals. Etanercept, a soluble TNF-α receptor fusion molecule (sTNFR), works by binding with the endogenous TNF-α, which subsequently is unable to interact with its native receptor. To ensure that etanercept reduced local TNF-α activity in the lung tissues, functional TNF-α activity and levels were measured by the WEHI assay [20] and ELISA respectively (Figure 1). As expected, compared to untreated mice, the TNF-α activity increased in the TB treatment arm. This increased TNF-α activity, was significantly reduced by the administration of adjunctive etanercept (p≤0.03) (Figure 1A, B). Moreover, to ensure that the TNF-α activity measured using the WEHI assay was indeed due to TNF-α and not other endogenous factors present in the sample, lung homogenates from both the standard treatment (RHZ alone) and adjunctive etanercept (RHZ plus etanercept) arms were pre-incubated with increasing concentrations of a neutralizing anti-murine TNF-α antibody and tested using the WEHI assay (Figure 1C). Lung homogenates from the adjunctive etanercept arm had consistently lower TNF-α activity, which remained unaffected by pre-incubation with the neutralizing antibody. However, TNF-α activity in the lung homogenates from the standard treatment arm decreased in a dose-dependent manner with increasing concentrations of the neutralizing antibody, with activities at 100 or 10 fold dilutions reaching levels observed in the adjunctive etanercept arm (p>0.098).

### Adjunctive Etanercept Increases Bacterial Clearance During the Continuation Phase of TB Treatment

**Figure 2:**
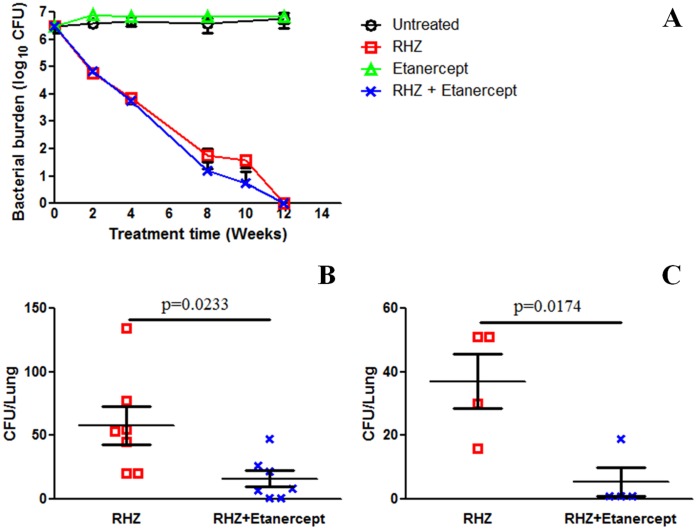
Bacterial burden in the lungs of mice. Six weeks after an aerosol infection with *Mycobacterium tuberculosis*, C3HeB/FeJ mice were split among four treatment groups: Untreated (no treatment), standard TB treatment (RHZ), etanercept alone and standard TB treatment with adjunctive etanercept (RHZ + Etanercept). The number of viable bacteria in the lungs were estimated by determining colony-forming units (CFU). Results are shown for the duration of study (panel A) and also as individual dot plots for 8 (panel B) and 10 (panel C) weeks after starting TB treatment. Compared to standard TB treatment, the addition of etanercept resulted in a significantly lower pulmonary bacterial burden, corresponding to the phase when a significant proportion of bacteria are multiplying slowly (panels B, C). Results are presented as mean (±SD) CFU in the lungs, detected from a minimum of four mice at each time point and for each group. CFU are presented on a logarithmic scale (log_10_) in panel A and on a linear scale for panels B and C and represent the same data.

The addition of etanercept did not alter bacterial killing significantly during the initial phase of TB treatment, when the majority of bacteria are replicating actively, and effectively cleared by antimicrobial treatment alone (Figure 2A). However, at later time-points (8, 10 weeks of treatment), corresponding to the phase when a significant proportion of bacteria are multiplying slowly (persisters), the addition of etanercept resulted in a significantly lower bacterial burden (p  = 0.0233; p  = 0.0174 at 8 and 10 weeks respectively) (Figure 2B, C). Both the groups receiving standard TB treatment or adjunctive etanercept were culture negative after 12 weeks of treatment.

Additional cohorts of mice were held for 12 weeks after cessation of treatment to assess for relapse. Only 10.5% (2/19) of mice receiving adjunctive etanercept versus 27.8% (5/18) receiving standard TB treatment alone relapsed.

### Adjunctive Etanercept Reduces TB-associated Pathology

**Figure 3:**
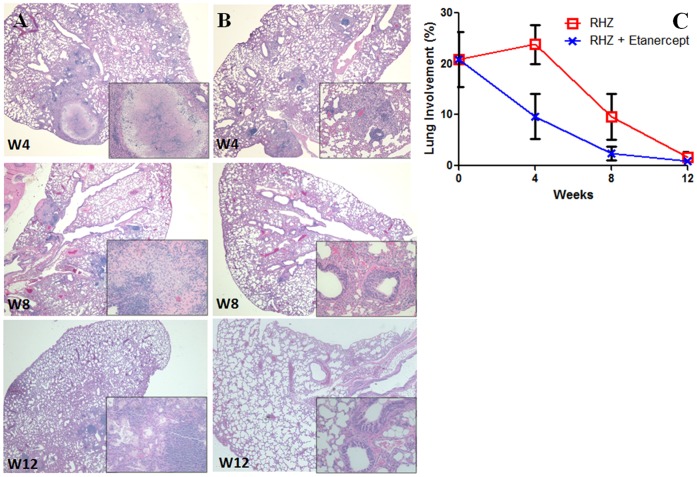
Adjunctive etanercept reduces TB-associated pathology. Hematoxylin and eosin staining (20x and 200x – inset) was performed on lung tissues. Images shown are representative of sections obtained from 4 animals per group. Each time-point is shown in weeks on the individual images. Lung pathology in animals receiving adjunctive etanercept (RHZ plus etanercept) (panel B) appeared to resolve earlier than those receiving standard treatment (RHZ alone) (panel A). Morphometric analysis confirmed these findings and demonstrated reduced lung involvement in mice treated with adjunctive etanercept (RHZ plus etanercept; blue x) versus standard treatment (RHZ alone; red □) (panel C). Results are represented as percentage lung involvement, calculated using ImageJ software.

Previous work using TNF-α inhibition, in mice demonstrated a role for TNF-α in granuloma formation and integrity. Mice receiving TNF-α inhibitors were reported to form lesions which were more loosely packed and lacking epithelioid cells [4]. To determine the effect of TNF-α inhibition on lung pathology, hematoxylin and eosin stains were assessed throughout the study. As shown in Figure 3, pulmonary lesions in animals receiving adjunctive etanercept (RHZ plus etanercept, Panel B) appeared to resolve earlier than those receiving standard treatment (RHZ alone, Panel A). Morphometric analysis also demonstrated reduced lung involvement in mice treated with adjunctive etanercept (RHZ plus etanercept) versus standard treatment (RHZ alone) (Figure 3C). These differences were statistically significant at 4 weeks (p = 0.0139).

### Immune Responses During TB Treatments

**Figure 4:**
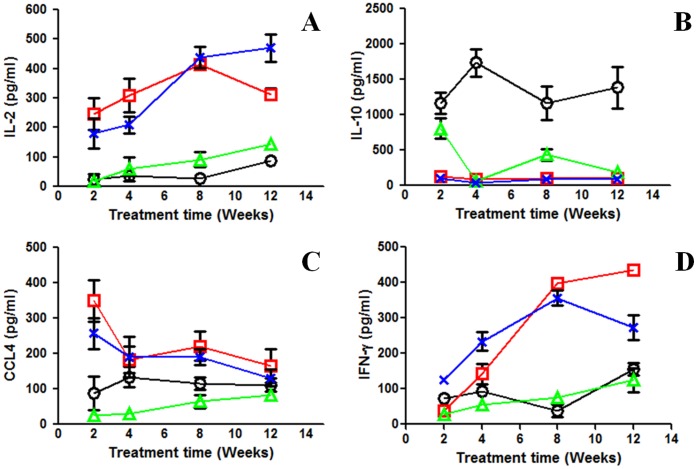
Immune responses during TB treatments. Splenic recall assays were performed to monitor the immune responses during TB treatment in animals receiving adjunctive etanercept (RHZ plus etanercept) (blue x) and standard treatment (RHZ alone) (red □). Infected but untreated mice (black ○) and mice treated with etanercept alone (green Δ) were used as controls. High levels of IL-2, IFN-γ and CCL4 were noted in mice receiving standard TB treatment with or without adjunctive etanercept. Conversely, higher levels of IL-10 were only noted in the untreated mice. Overall, this represents a transition, from a regulatory to an effector phenotype, following the onset of treatment. Determinations were made from four mice, and each assay was performed in triplicate. Results are expressed as mean level (pg/ml) (±SD).

Splenic recall assays were performed to monitor the immune responses during TB treatment with and without adjunctive etanercept. Infected but untreated mice and mice treated with etanercept alone were used as controls. High levels of IL-2, a cytokine required for development and differentiation of CD8+ memory T-cells, and CCL4, a chemokine produced in large quantities by CD8+ T-cells, were noted in mice receiving standard TB treatment with or without or adjunctive etanercept (Figure 4). Conversely, higher levels of anti-inflammatory cytokine IL-10 were only noted in the untreated mice. IFN-γ levels were also measured by ELISA. Similar to IL-2, IFN-γ levels increased progressively during TB treatment with RHZ alone or with adjunctive etanercept. However, neither untreated or etanercept alone treated groups produced appreciable levels of IFN-γ. Overall, this is indicative of a transition, from a regulatory to an effector phenotype, following the onset of treatment.

## Discussion

New and shorter TB regimens are needed to improve the treatment of TB and to support global control efforts. However, current TB treatments largely target actively replicating bacteria. Killing of the slow-replicating, persistent microbes requires protracted treatment with multiple drugs. Human studies demonstrate that patients receiving TNF-α inhibitors for chronic inflammatory diseases (rheumatoid arthritis, Crohn disease) can develop reactivation TB disease [7], [8], [18]. Adjunctive TNF-α inhibition in combination with effective TB treatment may therefore enhance the clearance of persisters, and could therefore potentially shorten TB treatments. Amongst all the animal models, multi-drug TB chemotherapy is most well established in the mouse. However, necrosis and hypoxia, key pathological features of human TB lesions, thought to favor survival of persistent bacteria, are lacking in conventional mouse strains. We therefore utilized C3HeB/FeJ mice, which develop well-organized and hypoxic TB granulomas, with central caseous necrosis [16], and evaluated whether adjunctive TNF-α inhibition could hasten bacterial clearance. We studied the time-specific inhibition of TNF-α, after granuloma formation had already occurred, a situation analogous to what would be seen with human patients presenting with active TB. Our data suggests that time-specific inhibition of TNF-α in combination with effective TB treatment, resulted in reduced necrosis and faster resolution of TB lesions. This was associated with improved bacterial killing during the continuation phase of treatment. The availability of mouse specific reagents also allowed us to evaluate the immune responses during TB treatments in this model. Since etanercept is a foreign antigen for mice, antibodies against the molecule are likely to appear following repeated administration of the protein. Lories RJ *et al* have shown that even though high antibody titers may be present in the serum of etanercept-treated mice, the presence of antibodies does not block the effect of etanercept [21]. To ensure that etanercept reduced local TNF-α activity in the lung tissues, we measured functional TNF-α activity by the WEHI assay [20] and total TNF-α protein by ELISA. As demonstrated previously in humans [10], TNF-α activity increased shortly after initiation of standard multi-drug TB treatment in our model. This increase in TNF-α activity was abrogated by the administration of adjunctive etanercept. Finally, to ensure that the TNF-α activity measured using the WEHI assay was indeed due to TNF-α and not other endogenous factors present in the sample, lung homogenates from both the standard treatment (RHZ alone) and adjunctive etanercept (RHZ plus etanercept) arms were pre-incubated with increasing concentrations of a neutralizing anti-murine TNF-α antibody and tested using the WEHI assay. These assays proved that the TNF-α bioactivity measured using the WEHI cells was indeed related to TNF-α and not to other cytotoxic factors. Moreover, splenic recall assays suggested that a predominant regulatory phenotype existed before the administration of TB treatments. However, standard TB treatment (with and without adjunctive etanercept) induced a switch to an IFN-γ+, IL-2+, CCL4+ effector phenotype indicative of strong Th1and CD8+ response. This is in keeping with human data by Caccamo *et al.* which demonstrated an increased CD8+ T-cell population in the blood of actively infected individuals following TB treatments [22]. Also, these data indicate that adjunctive use of etanercept with TB treatments did not prevent mice from generating a cytotoxic T-cell response. Though more generalized immune modulation in combination with antimicrobial therapy may also effect better bacterial killing [23], use of specific immunomodulators could reduce the untoward side effects. It should be noted that recent studies in mice [24], [25] and rabbits [26], using phosphodiesterase inhibitors, which modulate TNF-α, has also demonstrated improved pathology, and increased bacterial killing when combined with INH monotherapy. Finally, it should be noted that we utilized etanercept (sTNFR) in the current study due to its lower risks in causing reactivation disease than with TNF antibodies [18], [19]. However, given the proposed mechanism of action (effects on granuloma integrity), it is possible that greater therapeutic benefit could be achieved using TNF antibodies.

In summary, the current study provides proof-of-principle that modulation of TNF-α activity can hasten bacterial clearance when combined with standard multi-drug TB treatment. Moreover, oral TNF-α inhibitors such as tofacitinib (CP-690550) and fostamatinib are currently being evaluated for treating chronic inflammatory diseases [27], [28]. As oral drugs (versus currently available injectable TNF-α inhibitors), these agents are better suited (lower costs and ease of administration) for combination with TB programs in resource limited settings. Oral agents that modulate TNF-α should therefore be considered as adjunct therapies that could shorten TB treatments and will be the focus of our future pre-clinical studies. However, due to the multitude of human studies demonstrating that patients receiving TNF-α inhibitors for chronic inflammatory diseases (rheumatoid arthritis, Crohn disease) can develop reactivation TB disease [7], [8], [18], we acknowledge the significant risks of such treatment. Therefore, additional studies need to be performed to evaluate safety, before TNF-α inhibitors are used for TB treatment in humans.

## References

[1] Dye C, Williams BG (2008). Eliminating human tuberculosis in the twenty-first century.. J R Soc Interface.

[2] Haapanen JH, Kass I, Gensini G, Middlebrook G (1959). Studies on the gaseous content of tuberculous cavities.. Am Rev Respir Dis.

[3] Blumberg HM, Burman WJ, Chaisson RE, Daley CL, Etkind SC (2003). American Thoracic Society/Centers for Disease Control and Prevention/Infectious Diseases Society of America: treatment of tuberculosis.. Am J Respir Crit Care Med.

[4] Flynn JL, Goldstein MM, Chan J, Triebold KJ, Pfeffer K (1995). Tumor necrosis factor-alpha is required in the protective immune response against Mycobacterium tuberculosis in mice.. Immunity.

[5] Senaldi G, Yin S, Shaklee CL, Piguet PF, Mak TW (1996). Corynebacterium parvum- and Mycobacterium bovis bacillus Calmette-Guerin-induced granuloma formation is inhibited in TNF receptor I (TNF-RI) knockout mice and by treatment with soluble TNF-RI.. J Immunol.

[6] Kindler V, Sappino AP, Grau GE, Piguet PF, Vassalli P (1989). The inducing role of tumor necrosis factor in the development of bactericidal granulomas during BCG infection.. Cell.

[7] Keane J, Gershon S, Wise RP, Mirabile-Levens E, Kasznica J (2001). Tuberculosis associated with infliximab, a tumor necrosis factor alpha-neutralizing agent.. N Engl J Med.

[8] MMWR (2004). Tuberculosis associated with blocking agents against tumor necrosis factor-alpha–California, 2002–2003.. MMWR Morb Mortal Wkly Rep.

[9] Paige C, Bishai WR (2010). Penitentiary or penthouse condo: the tuberculous granuloma from the microbe’s point of view.. Cell Microbiol.

[10] Bekker LG, Maartens G, Steyn L, Kaplan G (1998). Selective increase in plasma tumor necrosis factor-alpha and concomitant clinical deterioration after initiating therapy in patients with severe tuberculosis.. J Infect Dis.

[11] Wallis RS, van Vuuren C, Potgieter S (2009). Adalimumab treatment of life-threatening tuberculosis.. Clin Infect Dis.

[12] Wallis RS (2005). Reconsidering adjuvant immunotherapy for tuberculosis.. Clin Infect Dis.

[13] Blackmore TK, Manning L, Taylor WJ, Wallis RS (2008). Therapeutic use of infliximab in tuberculosis to control severe paradoxical reaction of the brain and lymph nodes.. Clin Infect Dis.

[14] Wallis RS, Kyambadde P, Johnson JL, Horter L, Kittle R (2004). A study of the safety, immunology, virology, and microbiology of adjunctive etanercept in HIV-1-associated tuberculosis.. AIDS.

[15] Pan H, Yan BS, Rojas M, Shebzukhov YV, Zhou H (2005). Ipr1 gene mediates innate immunity to tuberculosis.. Nature.

[16] Harper J, Skerry C, Davis SL, Tasneen R, Weir M (2012). Mouse model of necrotic tuberculosis granulomas develops hypoxic lesions.. J Infect Dis.

[17] Hu YL, Kim HY, Kohno T, Khare SD (2007). Pharmacodynamic effects of the murine p75-Fc fusion protein in mice.. J Investig Dermatol Symp Proc.

[18] Wallis RS, Broder MS, Wong JY, Hanson ME, Beenhouwer DO (2004). Granulomatous infectious diseases associated with tumor necrosis factor antagonists.. Clin Infect Dis.

[19] Plessner HL, Lin PL, Kohno T, Louie JS, Kirschner D (2007). Neutralization of tumor necrosis factor (TNF) by antibody but not TNF receptor fusion molecule exacerbates chronic murine tuberculosis.. J Infect Dis.

[20] Espevik T, Nissen-Meyer J (1986). A highly sensitive cell line, WEHI 164 clone 13, for measuring cytotoxic factor/tumor necrosis factor from human monocytes.. J Immunol Methods.

[21] Lories RJ, Derese I, de Bari C, Luyten FP (2007). Evidence for uncoupling of inflammation and joint remodeling in a mouse model of spondylarthritis.. Arthritis Rheum.

[22] Caccamo N, Guggino G, Meraviglia S, Gelsomino G, Di Carlo P (2009). Analysis of Mycobacterium tuberculosis-specific CD8 T-cells in patients with active tuberculosis and in individuals with latent infection.. PLoS ONE.

[23] Cox JH, Ivanyi J (1988). The role of host factors for the chemotherapy of BCG infection in inbred strains of mice.. APMIS.

[24] Koo MS, Manca C, Yang G, O’Brien P, Sung N (2011). Phosphodiesterase 4 inhibition reduces innate immunity and improves isoniazid clearance of Mycobacterium tuberculosis in the lungs of infected mice.. PLoS ONE.

[25] Maiga M, Agarwal N, Ammerman NC, Gupta R, Guo H (2012). Successful Shortening of Tuberculosis Treatment Using Adjuvant Host-Directed Therapy with FDA-Approved Phosphodiesterase Inhibitors in the Mouse Model.. PLoS ONE.

[26] Subbian S, Tsenova L, O’Brien P, Yang G, Koo MS (2011). Phosphodiesterase-4 inhibition combined with isoniazid treatment of rabbits with pulmonary tuberculosis reduces macrophage activation and lung pathology.. Am J Pathol.

[27] Kremer JM, Bloom BJ, Breedveld FC, Coombs JH, Fletcher MP (2009). The safety and efficacy of a JAK inhibitor in patients with active rheumatoid arthritis: Results of a double-blind, placebo-controlled phase IIa trial of three dosage levels of CP-690,550 versus placebo.. Arthritis Rheum.

[28] Sweeny DJ, Li W, Clough J, Bhamidipati S, Singh R (2010). Metabolism of fostamatinib, the oral methylene phosphate prodrug of the spleen tyrosine kinase inhibitor R406 in humans: contribution of hepatic and gut bacterial processes to the overall biotransformation.. Drug Metab Dispos.

